# Variational Autoencoders for Network Lifetime Enhancement in Wireless Sensors

**DOI:** 10.3390/s24175630

**Published:** 2024-08-30

**Authors:** Boopathi Chettiagounder Sengodan, Prince Mary Stanislaus, Sivakumar Sabapathy Arumugam, Dipak Kumar Sah, Dharmesh Dhabliya, Poongodi Chenniappan, James Deva Koresh Hezekiah, Rajagopal Maheswar

**Affiliations:** 1 Department of Electrical and Electronics Engineering, SRM Institute of Science and Technology, Kattankulathur 603 203, Tamil Nadu, India; boopathc1@srmist.edu.in; 2 Department of Computer Science and Engineering, Sathyabama Institute of Science and Technology, Chennai 600 119, Tamil Nadu, India; princemary26@gmail.com; 3 Department of Electronics and Communication Engineering, Ashoka Women’s Engineering College, Kurnool 518 002, Andhra Pradesh, India; drsasivakumar@gmail.com; 4 Department of Computer Engineering and Applications, GLA University, Mathura 281 406, Uttar Pradesh, India; dipak.sah@gla.ac.in; 5 Department of Information Technology, Vishwakarma Institute of Information Technology, Pune 411 048, Maharastra, India; dharmesh.dhabliya@viit.ac.in; 6 Department of Electronics and Communication Engineering, Bannari Amman Institute of Technology, Sathyamangalam 638 401, Tamil Nadu, India; poongodi.me07@gmail.com; 7 Department of Electronics and Communication Engineering, Centre for IoT and AI (CITI), KPR Institute of Engineering and Technology, Coimbatore 641 407, Tamil Nadu, India

**Keywords:** data aggregation, energy optimization, data transmission, autoencoder, data compression

## Abstract

Wireless sensor networks (WSNs) are structured for monitoring an area with distributed sensors and built-in batteries. However, most of their battery energy is consumed during the data transmission process. In recent years, several methodologies, like routing optimization, topology control, and sleep scheduling algorithms, have been introduced to improve the energy efficiency of WSNs. This study introduces a novel method based on a deep learning approach that utilizes variational autoencoders (VAEs) to improve the energy efficiency of WSNs by compressing transmission data. The VAE approach is customized in this work for compressing WSN data by retaining its important features. This is achieved by analyzing the statistical structure of the sensor data rather than providing a fixed-size latent representation. The performance of the proposed model is verified using a MATLAB simulation platform, integrating a pre-trained variational autoencoder model with openly available wireless sensor data. The performance of the proposed model is found to be satisfactory in comparison to traditional methods, like the compressed sensing technique, lightweight temporal compression, and the autoencoder, in terms of having an average compression rate of 1.5572. The WSN simulation also indicates that the VAE-incorporated architecture attains a maximum network lifetime of 1491 s and suggests that VAE could be used for compression-based transmission using WSNs, as its reconstruction rate is 0.9902, which is better than results from all the other techniques.

## 1. Introduction

Wireless sensor networks (WSNs) have revolutionized the process of sensing data in various domains by comprising multiple sensor nodes. WSNs include sensors to detect environmental data and an analog-to-digital converter to convert analog signals into digital data. They also comprise a microcontroller or Raspberry Pi for processing data with the help of the stored machine learning (ML) or optimization algorithms. A communication module, like XBee, is also included for wireless transmission, along with a power source for supplying energy to the node. Therefore, WSNs can be placed in various remote and harsh environments where traditional wired sensors cannot be placed. Hence, WSNs are widely implemented in various fields, which include environmental monitoring, agriculture, healthcare, automation, surveillance, and military defense. The sensors included in WSNs create a distributed network that works to collect and transmit data to the source from its own base station. To achieve this, several wireless communication protocols are incorporated within the WSNs that permit the collection of information to be processed at the base station and enable several decisions to be made. The size, cost effectiveness, and flexible nature of WSNs make them suitable for various applications. However, their energy efficiency, network flexibility, and scalability are all factors that need to be improved in their present form.

Amongst their other limitations, energy efficiency occupies the highest priority, as it directly affects the overall performance of WSNs. The energy efficiency of WSNs can be improved by utilizing stored energy in an optimized way. Optimization methods are required, as WSNs are implemented with multiple sensors that can drain energy very quickly when all the sensors are in active mode. This can reduce the reliability of the system when it is implemented in the most important applications, such as healthcare and military surveillance, that necessitate continuous operation without any interruptions. Also, the draining of the energy supply from the battery may lead to the degradation of the WSN system, and so it is not suitable for long-term monitoring purposes. This limitation can be addressed by implementing an efficient network algorithm that can provide longer-term operation periods with a constant power supply. An efficient WSN model can also improve the costs spent on the monitoring system and protect the environment by reducing the number of batteries used and replaced.

### 1.1. Overview of Transmission Efficiency Improvement Methods

Traditional energy enhancement methods have optimized energy use by addressing the issues of signal interference, communication degradation, and bandwidth issues. Changing the characteristics of the carrier signal is the primary technique used for transmitting encoded data effectively to a destination with minimal energy requirements. Amplitude, frequency modulation, and phase shift keying are regular techniques that also provide reliable data communication between destinations that are great distances apart with minimal signal adjustments. Error control coding is also one of the traditional methods used to improve redundancy in WSNs in terms of the transmission of data to a destination by correcting errors.

**Figure 1:**
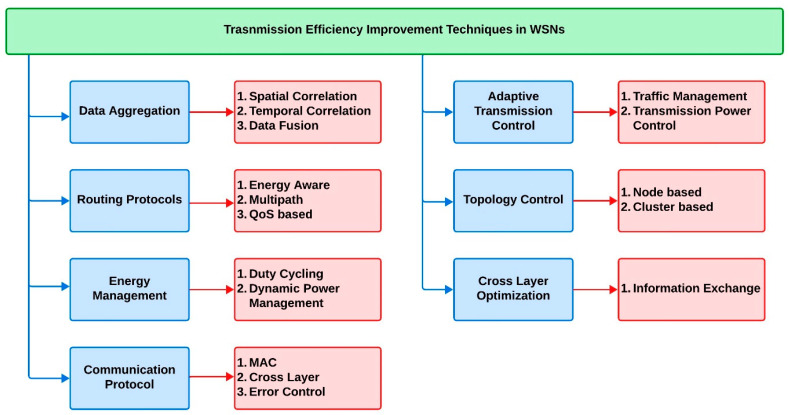
Transmission efficiency improvement techniques in WSNs.

The convolutional codes and hamming codes are some of the techniques that are widely used for such redundancy improvements. These methods improve the reliability of the data transmitted, even in noisy environments. Frequency- and time-based multiple access algorithms are methods that provide the simultaneous transmission of data from multiple sources to destinations connected to WSNs. Certain signal processing algorithms and equalization techniques have also been implemented in WSN systems to improve transmission efficiency. Equalization methods have been utilized in some applications where the transmission of data is dynamic due to channel conditions. Figure 1 indicates an overview of general transmission efficiency improvement techniques used for WSNs.

### 1.2. Variational Autoencoders

Variational autoencoders (VAEs) are a generative-model-based neural network approach that is widely used for unsupervised learning processes in data representation applications. The concept of VAEs is to extract the continuous latent information from the given high-dimensional data, like text, image, or any other signal, to provide compact data values that reflect the input data distribution. Unlike the regular autoencoders, the VAEs are included with a probabilistic framework based on a Gaussian distribution model that acts like an encoder, which allows for extracting the latent information effectively, in a distributed manner. Further, the output of the encoder is connected to a decoder network for resampling the distributed information received from the input. VAEs utilize an optimized loss function that regulates the reconstruction process on the latent space, and that improves the training process by learning only the meaningful information. Therefore, the performance of VAEs is comparatively better than that of the traditional autoencoders, as it provides scalable information. The reparameterization feature included in VAEs can provide gradient-based optimization from the useful information extracted from raw data. This interpretable ability makes VAEs suitable for big and complex data analysis. 

### 1.3. Motivation for Using VAEs in WSNs

The performance of WSNs is not satisfied in several applications, where it is required to operate with huge data generated by multiple sensors simultaneously. The performance is degraded due to limited energy availability, inefficient computational model, and reduced bandwidth allocation. These constraints can be addressed by implementing an efficient compression technique, and that reduces the size of data to be transmitted effectively. The nature of VAEs indicates that they can compress the information available on any data by preserving the important features. The encoding option of VAEs can provide the compressed sensor data in a lower dimension latent space, to enable reduced data communication, to achieve a better energy conservation on limited bandwidth resources. Moreover, the robustness and intelligence of a WSN model can be improved with VAEs, and that can result in anomalies, faults, and intrusion detection. The latent space representation of data in VAEs can provide privacy to the sensitive data that are used for communication. In general, the integration of WSNs with VAEs can improve the overall integrity of WSNs including energy efficiency, security, and fault tolerance. The motive of the proposed work is as follows:

- Provide an efficient compression technique for sensor data without losing valuable information;
- Improve the routing process for the transmission of data from the source to the destination;
- Enhance the energy efficiency of the overall sensor network to improve the network lifetime.

## 2. Literature Review

### 2.1. Energy Efficiency Improvement Techniques in WSNs

An improved grey wolf optimization technique [1] was developed to increase the energy efficiency of WSNs in industrial IoT applications. This improved model addressed the limitations of the traditional grey wolf method in terms of premature convergence and reduced population diversity. The simulation analysis indicated 333.51% betterment over the regular grey wolf optimization method on the network stability analysis [1]. A threshold-enabled scalable and energy-efficient scheme [2] was proposed to support the wireless networks that was implemented in a large-scale IoT scenario with around five or more different sensor nodes for regulating the data transmission process by avoiding unnecessary data transmission. It was achieved by implementing a hybrid threshold- based minimum cost cross layer transmission at every node to stabilize the energy saving mode. Hence, the model allowed data transmission only when it is necessary by understanding the static and dynamic factors of the connected sensor nodes at different stages. The simulation result indicated a betterment of 29% on energy efficiency and 68% of network lifetime enhancement over the traditional scalable and energy efficient scheme [2]. An alternative protocol approach [3] was designed to improve the energy efficiency by adjusting the time division and carrier sense multiple access in industrial wireless sensor networks. The work utilized carrier sense multiple access on light-weight data transmission and a modified selective activation technique for energy optimization and interference reduction between the redundant nodes. The work minimized the end-to-end delay and reduced the lower packet loss up to 25% on heavy traffic conditions when compared to the traditional industrial wireless network model implemented with IEEE 802.15.4 [3]. 

An advanced distributed energy-efficient clustering technique [4] was structured to improve energy efficiency in the autonomous cellular networks. The work transfers the data information in a better way through heterogeneous contexts with a 19% improvement on throughput over the LEACH [4]. A hybrid particle swarm optimization technique was incorporated with improved low-energy adaptive clustering hierarchy [5] for improving the energy efficiency by determining a best cluster head and corresponding cluster nodes. The simulation work indicated an improvement of 28% in energy efficiency and 55% in network lifetime over the previous methods [5]. A collaborative energy-efficient routing protocol [6] was designed for making a sustainable network model in the 5G and 6G communication systems. The work utilized the reinforcement learning approach for making a reliable cluster network for providing a successful transmission with the available energy. The multi-objective improved seagull algorithm was also included in the work for optimizing the overall performances. The experimental work indicated an improved energy efficiency of 50% over the previous methods [6].

### 2.2. Machine Learning in WSNs

A refined version of the Levenberg–Marquardt neural network [7] was proposed to improve energy efficiency by finding anomalies in WSNs. The work was also equipped with LEACH and energy-efficient sensor routing for making a reliable network connection between the source and the destination. The experimental work indicated a better performance with the model included with the machine learning approach over the regular methods [7]. An energy-efficient machine learning-based regression algorithm was included with the fuzzy based intelligent routing approach [8] for improving the energy efficiency in WSNs. A fine cluster head was identified in the work, through a fuzzy interference system using the hop-count method. The experimental analysis indicated a betterment in terms of packet delivery ratio and throughput with energy efficiency [8]. A reinforcement-learning-based multi-objective routing approach [9] was proposed with dynamic objective selection algorithm for optimizing energy consumption in IoT networks. The algorithm utilized informative-shaped rewards with correlated objectives for an efficient learning process. The work indicated an excellent delivery ratio along with a perfect data delivery latency [9].

An artificial-neural-network-based approach was implemented [10] to select a better cluster head from the connected WSNs. The artificial neural network algorithm was equipped to analyze residual energy, number of nearby nodes, and distance to the base station for selecting the best cluster head. The algorithm was also included with energy-efficient clustering algorithm for minimizing the energy consumption. It was achieved by switching OFF the cluster heads and their corresponding nodes, when there is no signal received for transmission, for a certain duration. The experimental work indicated a betterment in terms of energy enhancement and network lifetime over the traditional methods [10]. An enhanced energy optimization approach was structured with a machine learning technique [11] to improve the energy efficiency in industrial wireless sensor networks. A knowledge-based learning technique was included in the work to detect the sensor node, one that can deliver the data information with minimum energy usage. The model also had a feedback control system for estimating the best routing process in the network. Hence, the work reduced the network traffic and improved the transmission energy consumption to 64.72% when compared to the regular methods [11]. A multi-agent-based reinforcement learning approach was designed [12] to predict best energy efficient routing in WSNs. The work reduced the transmission delay and thus improved the average latency and network lifetime [12]. A novel self-driven continual learning framework [13] was designed for motor fault diagnosis. The model can detect faults in WSN by making considerable customizations [13].

### 2.3. Autoencoders in WSNs

An error bond mechanism was incorporated to the autoencoders [14] for making an image compression technique suitable for WSNs. The work minimized the redundancies from the input data to improve the energy efficiency through exploitation of spatial and temporal correlations. The experimental work indicated a better decoding efficiency over the previous method at the rate of 70%. The work attained an energy efficiency of 50% improvement, at a compression value of 38.6% [14]. An efficient resource allocation system was developed using autoencoders [15] for device-to-device communications. The approach was included with Hungarian algorithm, for reducing the data ambiguity in the data matrix. The experimental work achieved an accuracy of 83% and 90% in both training and validation, and the overall computational complexity was also found to be satisfied in the work, and that led to the minimum energy consumption [15]. A hybrid algorithm was generated using autoencoder and the traditional WinRAR [16] for making an efficient compression technique, for both numerical and image data transmission. The work utilized the attention layer of autoencoder to minimize the reconstruction error. The performance of the hybrid algorithm was verified with the openly available oceanic data and found satisfied with a better compression ratio of 69% over the WinRAR and 48% over the multilayer autoencoders [16]. An autoencoder- based machine learning technique [17] was proposed to improve the image transmission in the underwater IoT applications. The autoencoder method outperformed in underwater with its limited bandwidth. The work also overcomes the variable path loss in underwater scenario and provides a robust and efficient image transmission [17]. A VAE- based data compression technique was incorporated with the traditional CNN and Restricted Boltzmann Machine [18] for compressing the numerical temperature sensor data. The experimental result indicated an average reconstruction error value of 0.0678 °C with 95.83% of reduced energy consumption over the traditional methods [18]. Hence, it indicates that VAEs are widely preferred on wireless transmission for image compression applications over the numerical sensor data applications. 

### 2.4. Summary

The energy efficiency methodologies of WSN involves several techniques like routing, clustering, and data aggregation processes. Nature inspired and metaheuristic optimization methods were widely used for making such energy efficient algorithms. However, those optimization algorithms may not be suitable for problems of WSN and customizing such optimization algorithms have become a complex part. Also, the computational cost and the requirement of peripheral devices improve the energy utilization of such optimization algorithms. In some applications, an uneven energy utilization can be observed, and it may result in sudden network failure. Similarly, optimization-based data aggregation systems have chances for missing the useful information generated by the sensors. Hence the machine learning- based approaches are introduced in the recent years to improve the energy efficiency of WSNs. Machine learning methodologies are designed to improve energy efficiency through anomaly detection, intelligent routing, cluster head selection, and data compression. It is achieved by leveraging adaptive learning and data-driven insights on WSN. Analyzing data and energy consumption patterns also improve the performance of WSN by predicting the energy shortages in advance. K-means clustering and fuzzy logic systems seem to be the popular algorithms that are incorporated in ML- based WSNs for optimizing the cluster head selection process. The neural network-based approaches improve the latency by observing the real-time conditions and data transmission patterns simultaneously. However, the ML algorithm might consume more energy than traditional optimization approaches, because of its nature on analyzing multiple data at the same time.

The proposed work aims to address the energy consumption issues of the previous methods, by using a data compression technique. The traditional optimization and ML- based algorithms are structured to analyze a huge amount of data for taking certain decisions, like routing path or cluster head selection. Therefore, the computational complexity of such models may take an additional consumption of energy for the operation of such complex algorithms. The proposed work addresses this issue by implementing an intelligent data compression technique using a popular ML technique called Variational Autoencoders. In traditional works, the huge sensor data were considered only for analysis; whereas in the proposed work, the huge sensor data is compressed directly, and it results in the minimal data transmission, to save the energy consumption on data transmission. VAEs were originally designed for compressing image data transmission, and it is customized in this work for compressing the numerical sensor data by adjusting their encoder and decoder properties. This adjustment allows the VAEs to observe numerical input features with an optimal compression and reconstruction accuracy. 

## 3. Methodology

### 3.1. Autoencoders vs. Variational Autoencoders

**Figure 2:**
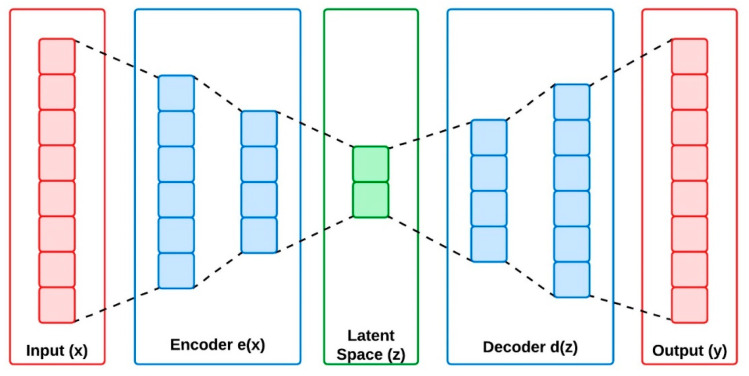
Architecture of autoencoders.

**Figure 3:**
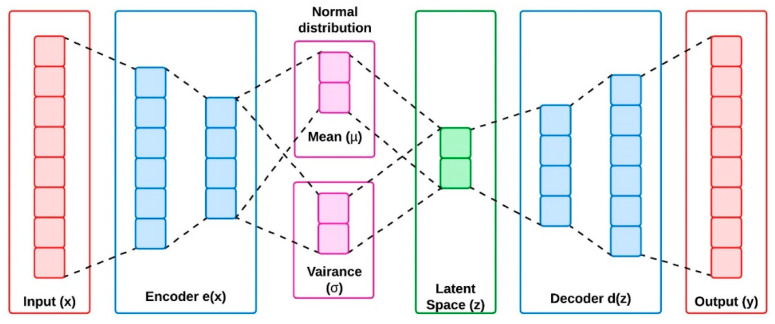
Architecture of variable autoencoders.

Data compression is the method widely used in applications where the dimensionality of the primary data must be reduced. The motive of such techniques is to share the same amount of information with minimum transmission bits. The autoencoders and variational autoencoders are the basic approaches implemented for data compression in machine learning- based applications. This approach transforms the data from higher to lower dimensional space through compression technique. Autoencoders can learn information efficiently from the unlabeled data through its encoder and decoder blocks. The encoder compresses the data from higher dimensional space to latent space, and the decoder is implemented to convert the latent space information back to higher dimensional space. However, the autoencoders have the limitation of generating valid information from the non-regularized input data. Variational autoencoders were developed to address this issue by improving its generative capability on the whole latent space created from the encoders. The encoders of VAE create output parameters with a predefined distribution for its every input created from the latent space. To regularize the latent space, VAEs are imposed with a constraint on the latent distribution, making it to provide a normal distribution [19]. Figure 2 and Figure 3 represent the architectural overview of autoencoder and the variational autoencoders.

### 3.2. Architecture of Variational Autoencoders

VAEs are deep latent space generative models that exhibit excellent results on various applications like language models, protein design, and image generation. The VAEs are found to be one of the successful approaches among the other unsupervised learning techniques. The basic idea of VAE is to create a meaningful data distribution from the encoded information [20,21]. In simple way, it can be descripted as: VAEs are utilized to generate a new data sampling from the given input information. For this, the VAEs are equipped with three major blocks called encoder, latent space and decoder. Encoder is the primary block that is used to compress the input information for making it suitable for the latent space. The latent space receives the compressed information from the encoder through the normal distribution with mean (µ) and variance (σ), and reduces the dimensionality of the received data by preserving the major and novel information available in the input. Decoder is the final block that reconstructs the information available in the latent space to its original form, same as of the input.

The Autoencoders (AEs) learn the features extracted from the compressed data structures of the input, and decompress the data structures again, to provide a valid output. In contrast, VAEs utilize the Bayesian model for extracting the features from the compressed data structures of the input to generate feature parameters through probability distribution of the given input. From this, the VAEs can create a new form of input data. Hence, the VAEs are represented as a generative architecture and AEs are represented as a reconstruction architecture. In VAEs, the actual input $x=x_{i=1}^{N}$ generates a random sample $y=y_{i=1}^{N}$ through discrete or continuous distribution variable. In high dimensional space, the output (y) provides a random vector from the latent space (z) generated from the encoder output e(x).

VAEs are included with a datapoint (x) as their input to their neural network block called the encoder, where its output is presented as a latent space (z). The weights and biases of the latent space (z) are adjusted with normal distribution parameters of mean (µ) and variance (σ). The latent space (z) is further connected as an input to the decoder block, where the neural network included in the decoder block provides an outcome as probability distribution of the given input. The Kullback–Leibler (*KL*) divergence is utilized to give an approximate distribution for the decoder d(z) with some e(x) as shown in Equation (1). Baye’s rule is applied to Equation (1) for reducing the *KL* divergence as shown in Equation (2).

(1) $$D_{KL}\;[\;e\left(x\right)\;||\;d\left(z\right)\;]=\sum_{z}e\left(x\right)\log\left[\frac{e\;(x)}{d\;(z)}\right]=E\;\left[\log\left[\;e\left(x\right)-d(z)\right]\right]$$

(2) $$E\;\left[\log\left[\;e\left(x\right)-d(z)\right]\right]=E\;\left[\log e\left(x\right)-\log d\left(z\right)-\log(e)-\log\left(d\right)\right]$$

Since the *KL* divergence is expected to be shown with respect to z, the Equation (2) can be further presented as Equation (3) and the $D_{KL}$ is concluded as Equation (4) as it is always a positive one.

(3) $$E\;\left[\log e\left(x\right)-\log d\left(z\right)-\log(e)-\log\left(d\right)\right]=log\;\left(d\right)-\left[E\left[\log d(z)\right]-D_{KL}\left[e\left(x\right)||\;d\right]\right]$$

(4) $$\log d\;\geq E\left[\log d(z)\right]-D_{KL}\left[e\left(x\right)||\;d\right]$$

Evidence Lower Bound (ELBO) of VAEs is shown in Equation (4), and it is represented as the loss function of the neural network on its training process. The $E\left[\log d(z)\right]$ is presented as a reconstruction function, that is, the resultant of latent space (z). $D_{KL}[e\left(x\right)||d\left(z\right)]$ is used to find the similarity between the latent space (z) and its target distribution d(z). Therefore, there will always be two components on Equation (4), that makes the output similar to the input, by making latent space (z) distribution similar to the target distribution d(z) as much as possible.

### 3.3. VAEs for WSNs

In the proposed work, the VAEs are utilized for compressing WSN data, using a neural network architecture with the help of an encoder and decoder. The encoder is placed on the source to compress the generated data from the sensor nodes as a lower dimensional latent space, while the decoder is placed on the destination to reconstruct the received data into its original value. Therefore, the property of the encoder in VAE is modified in the proposed work with a fully connected layer, for making the model suitable for reading the numerical inputs from the dataset with reduced power consumption, and the ReLU activation function is included for improving the learning stability with the limited computational resources. It is achieved by preventing the overfitting issues on the learning process. Similarly, a well distributed latent space is required for reducing the reconstruction errors of VAE, and that is achieved by fine tuning the model parameters. The latent dimensionality and weights on the reconstruction and *KL* divergence loss are customized in this work, to make VAE suitable for the proposed application. The performance of the trained VAE can be improved by having a continuous optimization or fine-tuning process while implemented on the real time WSN architectures. This iterative approach improves the performance of VAE, based on the feedback received from the actual network. The energy utilization of the VAE can also be improved by this iterative approach, as it operates the essential data with minimal resources. The performance of the proposed model is also improved on diverse and variational data patterns, as the network is retrained regularly by varying the hyperparameters of VAE. The integration of VAE to WSN provides a better lifespan on WSNs, as it reduces the amount of data transmission through compression technique. The compression remains effective as VAE preserves the crucial information on the generated sensor data. Similarly, VAE improves the data processing ability of the WSNs that results in effective decision making and analysis on the data transmission process. Overall, the proposed work is found to be suitable for WSN applications that are implemented with limited resources. The algorithmic flow of VAE in WSN is descripted below in Algorithm 1.
**Algorithm 1.** Algorithmic flow of VAE in WSN 1: Input Sensor data (*x*)2: VAE Architecture Design the encoder *e*(x) and decoder (*y*) neural networks for WSN applications Indicate the latent dimensionality of the VAE (*z*)3: Encode the Sensor Data (*x*) Get the mean (µ) and log variance (*σ*) of the latent space (*z*) representation through the encoder *e*(*x*)(µ, *σ*) = *e*(*x*)4: Sample from Latent Space (*z*) Sample the mean (µ) and log variance (*σ*) to obtain the latent representation (*z*) through a normal distribution*z* ~ *N*(µ, *σ*), *where σ* = *exp*(0.5 × *logσ*)5: Decode Latent Representation Pass the latent representation (*z*) through the decoder *d*(*z*) to reconstruct the sensor data:*y* = *d*(*z*)6: Loss Calculation Calculate the reconstruction loss from the original input *x* and the reconstructed data *x_recon*, and calculate the *KL* divergence loss to regularize the latent space distribution:*reconstruction_loss* = −*Σ*(*x* × *log*(*y*)) + (1 − *x*) × *log*(1 − *y*))*KL_divergence_loss* = −0.5 × *Σ*(1 + *σ* − µ^2^ − *exp*(*σ*)) 7: Total Loss Calculation: Combine the reconstruction loss and the *KL* divergence loss to form the total loss:*total_loss* = *reconstruction_loss* + *KL_divergence_loss*8: Optimization and iteration: Backpropagation is used to minimize the total loss and iterate over the dataset for multiple epochs, adjusting model parameters.9: OutputCompressed representation (*z*) and reconstructed data (*y*)

## 4. Experimental Setup and Results

The performance of the proposed work is verified with the dataset collected from the Intel Berkeley Research Lab [22]. The dataset contains temperature, light, humidity and voltage readings collected from 54 sensors that are placed in the lab for 38 days. Data was collected continuously for every 31 s from the lab with the help of a TinyOS platform. The missing values were normalized in the dataset through a mean average method, and the sensor values were regularized with Min-Max scaling function to ensure the uniformity among the connected sensors. The preprocessed dataset is further augmented to improve the training performance by increasing the sample sizes on the available dataset. The augmented dataset is divided into two sets with a ratio of 80% (40,000 samples) and 20% (10,000 samples) for the training and testing process in VAE integrated WSN architecture. 

**Figure 4:**
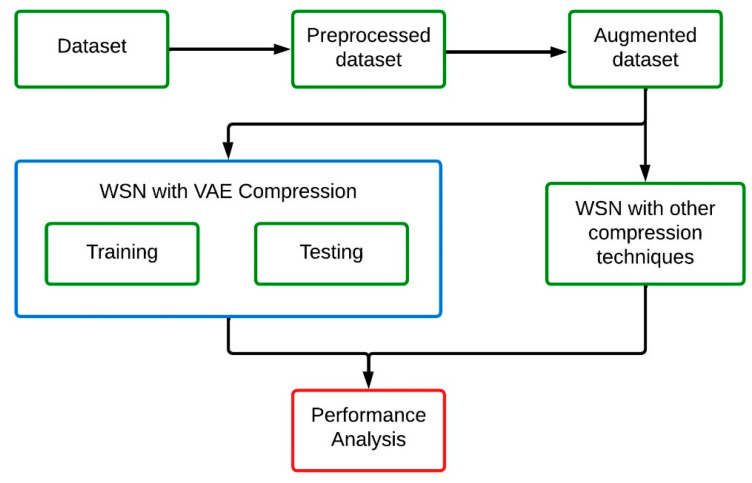
Experimental flow of the proposed work.

The simple workflow of the experimental analysis is shown in Figure 4. The performance of the proposed work is compared to that of the Compressed Sensing (CS) technique [23], Lightweight Temporal Compression (LTC) [24], and the Auto Encoder (AE) technique [25]. Compressed sensing is a signal compressing technique that is basically used for non-adaptive linear measurements. It is included in WSN for minimizing the spatial and temporal correlations of the sensor data, and that can be reconstructed later based on the requirement. Implementing CS to WSN is quite simple than any other technique, as most of the sensor data are represented in sparse formats. CS approaches are familiar for its effectiveness on reconstruction of data from the minimum number of random linear measurements. The performance of the CS can be improved further when it is implemented with convex optimization. Lightweight temporal compression is a time series sensor data compression technique used for reducing the transmission overhead. LTC stores only the changes on the transmitting data rather than storing them all together. This minimizes the amount of data transmitted between the source and destination, by preserving the major temporal trends and patterns of the sensor data. The encoding of LTC is simple, and that does not require any major computational facility. As the encoding is simple, the energy utilization of LTC is also minimal in nature. This makes the LTC well suited for several WSN applications. Autoencoder is an unsupervised learning approach for efficient data representation. AEs are best in reducing the reconstruction error, and that improves the compression efficiency. This makes AE suitable for complex WSNs.

To evaluate the performance of the proposed work, the VAEs are finetuned for the WSN application with customized encoder and decoder layers. As the dataset used for the work contains the information of 54 sensors, the input layer is structured with 54 different neurons for gathering the data in an efficient way. The hidden layers included between the input and the latent space layer were reduced gradually for observing the useful information from the given data. The neuron layers at the latent space are customized dynamically with respect to the feedback received from the output layer. Table 1 represents the number of neurons in each layer.

MATLAB R2023a simulation is utilized in the work for evaluating the performance of the proposed concept with the existing one. The output of the pretrained VAEs is incorporated to the WSN architecture for transmitting the compressed information, for estimating its performance on the transmission. Similar estimation is done for the other existing techniques, to prove the betterment of the proposed work. The network parameters used for analyzing the WSN performance is presented in Table 2.

### 4.1. Evaluation Metrics

The experimental work aims to analyze the compression efficiency of the VAE and is also experimented to present its betterment on transmitting data to the destination with minimal energy requirement. Compression ratio, and reconstruction quality are the factors considered in the work, to estimate the performance of VAE over the existing techniques. 

Compression ratio ($C_{R}$) defines the amount of original sensor data that has been compressed by the approach. A higher value of compression indicates its efficiency. Compression ratio can be descripted mathematically as follows.

(5) $$C_{R}=\frac{Original\;data\;in\;bytes}{Compressed\;data\;in\;bytes}$$

Reconstruction quality $\left(R_{Q}\right)$ is used to represent the accuracy of the reconstruction of data from the given compressed data. Mean Square Error (*MSE*) is the primary metric used for estimating the reconstruction quality. A higher reconstruction quality gives a minimal loss information of the given input.

(6) $$R_{Q}=1-\frac{MSE}{Variance\;of\;original\;data}$$

Energy consumption, delay and the network lifetime availability are the factors considered in the work for estimating the performance of VAE integrated WSN model. Energy consumption is calculated for the operation of the network in full load operation with respect to time. It represents the amount of energy required for an efficient operation, and that can be represented mathematically as follows.

(7) $$Energy\;consumption\left(E_{c}\right)=Power\times Time$$

Delay is used to analyze the time taken for transmitting a data from the source to the destination, and is denoted in milliseconds. It includes compression and decompression time along with the transmission time, and is represented as follows.

(8) $$Delay\left(D\right)=D_{compression}+D_{decompression}+D_{transmission}$$

Network lifetime is the parameter that can present the overall operation time of the WSN. It is directly related to the energy required for compression, transmission and the routing process. The below formula indicates the mathematical representation of the network lifetime.

(9) $$Network\;lifetime\left(N_{L}\right)=\frac{No.\;of\;nodes\times Given\;energy}{Avg.\;consumption\;per\;unit\;time\times Active\;nodes\;in\%}$$

### 4.2. Performance Analysis

**Figure 5:**
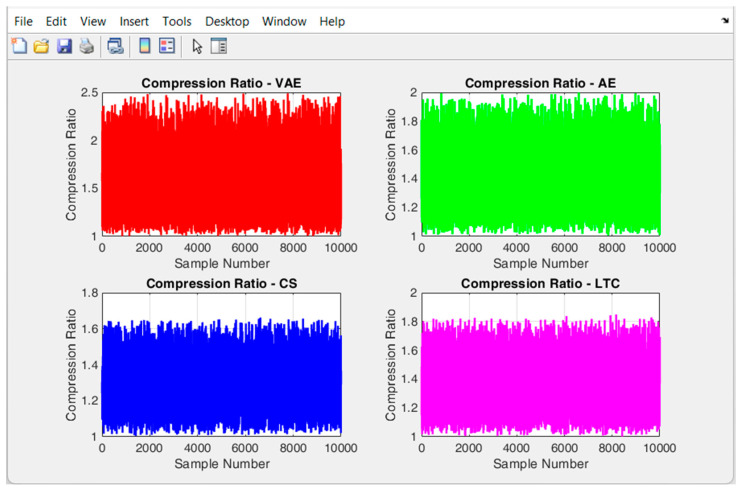
Comparison of the compression ratio of different methods.

The comparison chart of the compression ratio of the proposed VAE, in comparison to the existing methods is shown in Figure 5, and its average value is projected in Table 3. Figure 5 signifies that the compression ratio of VAE is comparatively higher than the three (AE, CS and LTC) existing methods. The maximum compression ratio of VAE reaches near 2.5, and its average compression ratio is 1.5572 which is better among all the existing methods considered. AE that attains an average compression ratio of 1.4111, provides a competitive compression ratio to the proposed VAE. CS algorithm provides the least average compression ratio of 1.2956, whereas the compression ratio by LTC is 1.3613. This analysis indicates that the amount of data transferred through VAE is comparatively less, than that of the other methods. Since the amount of data transferred though VAE based WSN is less, it can provide a better network lifetime than the other approaches. The performance of the VAE is found satisfactory, due to its probabilistic nature of encoding and decoding process. Similarly, the latent space regularization on the objective of VAE, makes the model learn the compact data representations at a high compression rate.

**Figure 6:**
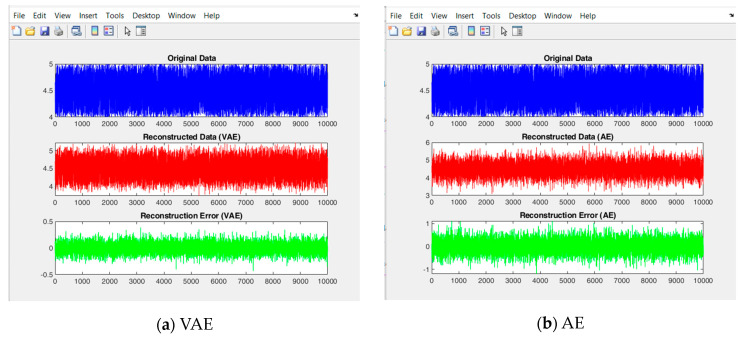

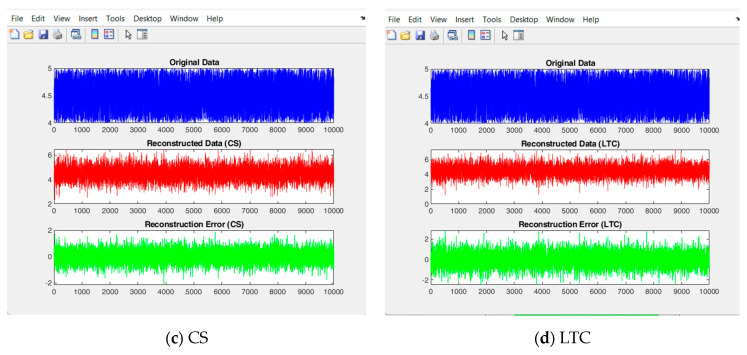
Comparison of reconstructed data and error of different approaches.

Compression ratio is one of the primary analyses that is required for calculating the amount of data transferred in a WSN. However, data reconstruction rate needs to be considered for identifying an overall efficiency of a compression algorithm. Figure 6 indicates the reconstruction data and its error value of the analyzed methods. It compares the reconstruction data of all 10,000 original data samples with the reconstructed data, to find the reconstruction error. The average reconstruction rate of each model is shown in Table 4. It can be found that the reconstruction rate of VAE is high at 0.9902, which shows a better reconstruction rate of 0.0793 than the AE model, due to the involvement of Kullback-Leibler divergence in VAEs. *KL* helps in avoiding the overfitting issues, and the loss function of VAE reduces its reconstruction error. LTC shows a poor reconstruction rate of 0.4984 and CS provides a decent reconstruction rate of 0.7552. From this analysis, it is observed that VAE provides excellent compression and reconstruction rate than the comparative methods. The experimental work identifies that CS algorithm provides a low compression rate and the LTC algorithm provides a poor reconstruction rate. 

**Figure 7:**
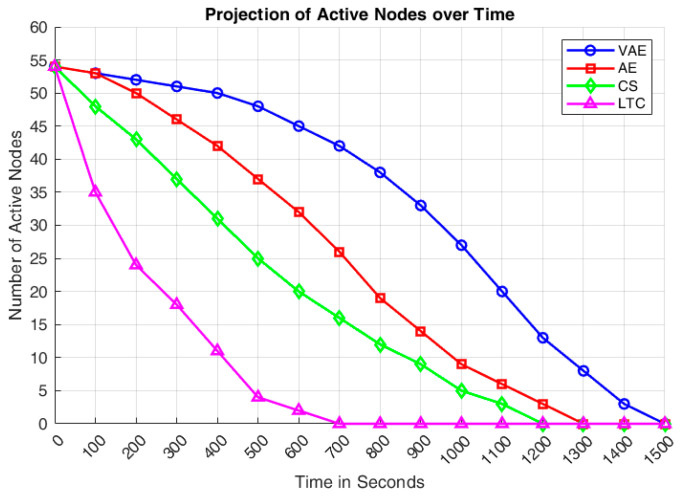
Comparison of active nodes over simulation time.

Figure 7 indicates the number of active sensor nodes available in the WSN architecture on every 100 s, in the overall simulation time of 1500 s. It can be discovered that the number of active nodes available in the VAE incorporated WSN is always higher than all the other methods. This has been attained as the number of retransmissions are less in VAE due to its betterment in reconstruction rate. The performance of AE comes in between VAE and CS, where all the active nodes are dead around 1300 s of simulation time. It has also been observed that the number of active nodes of both AE and VAE are same up to 100 s, then a steady drop has been observed from 100 to 800 s and that leads to all node dead state before 1300 s. Similarly, all the sensor nodes of CS and LTC approaches went into dead state before 1200 s and 700 s respectively. It has also been observed that VAE exhibits significantly better performance, with a notable deviation up to 1000 simulation seconds compared to the other three methods.

**Figure 8:**
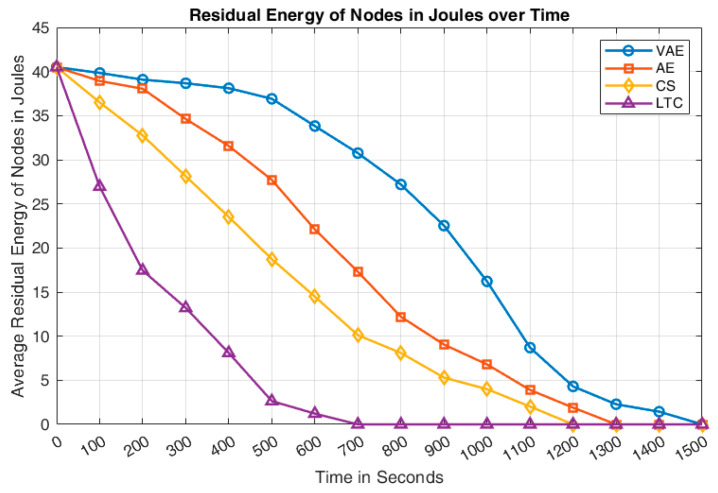
Comparison of residual energy over simulation time.

The residual energy availability of the sensor network for all the approaches has been shown in Figure 8. It can be deduced that the residual energy of VAE included sensor network is comparatively higher than that of the other approaches. It is achieved due to the better compression rate of VAE. Though the compression rate of AE is better than that of VAE, its poor decompression rate increases its total number of retransmissions, and that leads AE to perform lower than VAE in terms of residual energy. The performance of CS approach shows a steady energy drop from the beginning that leads to a complete network failure before 1200 s. The LTC indicates the poorest residual energy performance due to its poor compression rate. The poor compression rate makes the network to send huge amount of data and that allows the nodes to consume huge energy on its operation.

**Figure 9:**
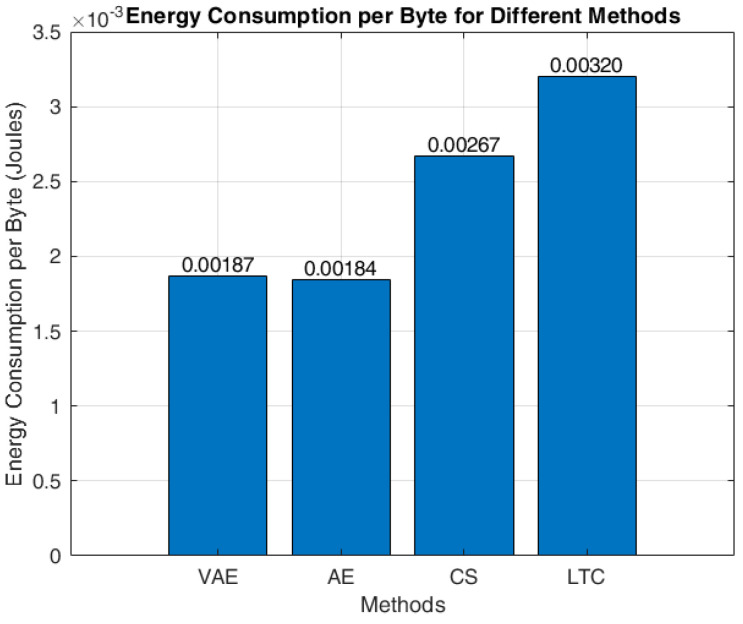
Energy consumption of different algorithms at per byte transmission.

Figure 9 specifies the energy consumption by the nodes for transmitting 1 byte of data. It can be determined that the AE approach consumes the least energy of 0.00184 joules for transmitting 1 byte of data, whereas VAE consumes a slightly higher energy of 0.00003 joules than AE, due to its superior data compression ratio. Hence, the total amount of data retransmission becomes lesser in VAE, and that enhances the network lifetime. LTC consumes very large energy of 0.00320 joules, as its computational cost is very poor among the other methods. CS is observed with an energy consumption of 0.00267 joules for its operation. In general, the overall energy consumption of a WSN architecture tends to increase with the number of sensor nodes deployed. This is because more sensor nodes generate more data, which can lead to network congestion. Network congestion, in turn, can result in a higher drop rate and more retransmissions. Consequently, the energy consumption per byte of transmission can potentially increase due to these factors. The specific energy consumption per byte provided for the current set of 54 sensor nodes has been given in Figure 9 and may vary with the addition of more nodes.

**Figure 10:**
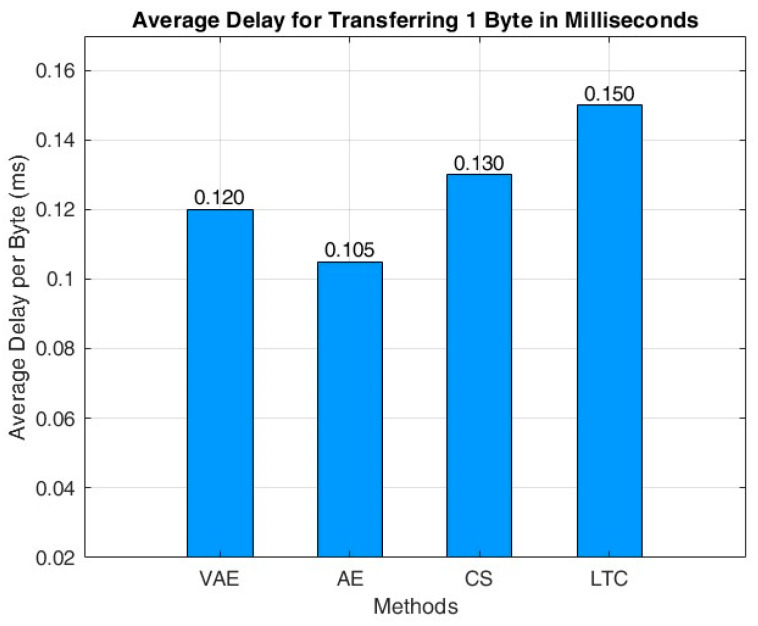
Average time taken for transferring 1 byte of data.

**Figure 11:**
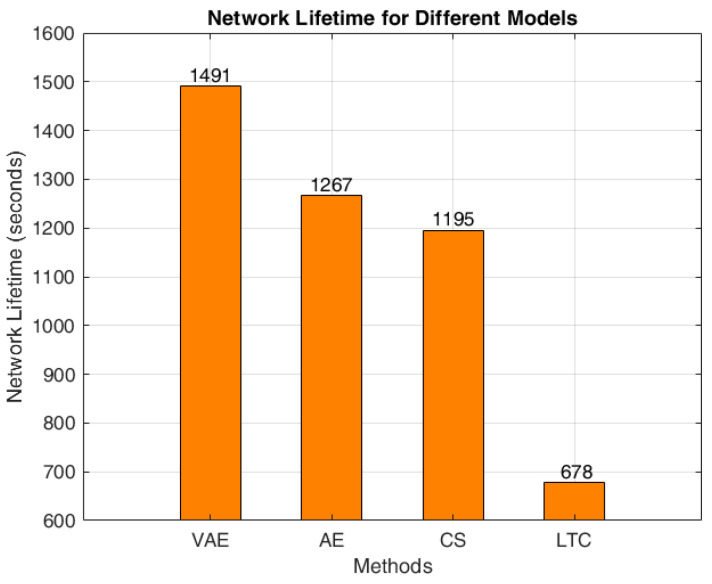
Network lifetime using different methods.

The average time taken for data transmission and the network lifetime of the proposed WSN architecture with various compression technique are shown in Figure 10 and Figure 11 respectively. It can be found that AE provides a minimum delay of 0.105 milliseconds for transferring 1 byte from source to destination. The delay of AE model is comparatively better than the VAE, as the compression rate is lower in AE. However, the better compression rate of VAE gives an overall improvement in the network lifetime that can be seen in Figure 11. The VAE attains a maximum network lifetime of 1491 s; whereas, in AE the network life is 1267 s, that is 224 s better than that of the AE. The transmission delay of both CS and LTC are slightly higher than that of the VAE, and their network lifetime is also comparatively lesser than that of the VAE. Moreover, the LTC shows the least network lifetime of 678 s.

### 4.3. Discussion

The performance of the proposed work was verified in a common network setup that is shown in Table 2 with the standard ODV routing with LEACH cluster head selection model. The performance of the proposed work can also be improved in terms of network lifetime, delay or energy consumption with an alternative routing or improved cluster head selection approach. However, the performance of the proposed work can be affected with several factors like hardware limitations, signal interference and power source availability on real-time deployment. Therefore, a careful consideration of computational components, memory units, and power resources are required for a successful ML- based implementation. Also, the complexity of the ML algorithm, number of connected sensors and the amount of data to be processed require a powerful CPU, GPU and memory units. For implementing the proposed work, a multi-core CPU of 3.0 GHz or higher is required for handling the coordination between the connected components. Similarly, a GPU of 8–12 GB and a RAM of 16–32 GB are required for processing the huge data generated by the sensors in parallel to the execution of the VAE algorithm without any buffering. In such cases, power consumption by the compression algorithm would be a constraint, and that can be rectified by implementing an efficient batch processing technique along with a careful thermal management process.

## 5. Conclusions and Future Research Directions

Network lifetime is one of the primary constraints considered for implementing a successful WSN. Several techniques like routing, clustering, load balancing, and duty cycling are the most widely used methods for WSN lifetime improvement. The proposed work utilizes a data aggregation method for improving the network lifetime, where the variational autoencoders are considered. To prove the efficiency of the variational autoencoders, its performance on data compression and reconstruction was compared to that of several existing methods. This work utilizes the Intel Berkeley Research Lab dataset for its analysis, which was found to be satisfied with VAE on both compression and reconstruction. Reconstruction plays a significant role in the satisfaction of any compression technique to reach the actual sensor data at the destination. The VAE-based method was found to be satisfactory, with a better network lifetime than all the comparative methods. However, the delay and energy consumption for data transmission seem to be slightly higher than that of AE. AE attains a minimum delay and a better energy consumption due to its lesser computational cost. Although AE is better on delay and energy consumption, it cannot overtake VAE, as its reconstruction rate is marginally higher than that of AE. In the future, the VAE network will be finetuned with customized neuron layers for reducing its computational complexity, to obtain a better efficiency on delay and energy consumption.

## Figures and Tables

**Table 1:** Neuron count in each layer of the proposed VAE network.

Parameter	Description	Value
Encoder	Input layers	54
	Hidden layer 1	128
	Hidden layer 2	64
	Latent space	16 (8 for mean and 8 for log variance)
Decoder	Latent input	8
	Hidden layer 1	64
	Hidden layer 2	128
	Output layer	54
Epochs	Training epochs	100
Batch size	Size of batch training	32
Learning rate	Optimization rate	0.01
Activation function	ReLU	
Loss function	Reconstruction + KL	
Optimization algorithm	Gradient Descent	

**Table 2:** Simulation parameters of WSN.

Parameters	Description
Network size	275 m × 275 m
Sensor node count	54
Given energy to each sensor nodes	0.75 joules
Packet size (data)	3000 bits
Packet size (control)	200 bits
Simulation time	1500 s
Routing	ODV
Cluster	LEACH
Communication Speed	250 kbps (IEEE 802.15.4)

**Table 3:** Average compression ratio of different methods.

VAE	AE	CS	LTC
1.5572	1.4111	1.2956	1.3613

**Table 4:** Average reconstruction rate of different methods.

VAE	AE	CS	LTC
0.9902	0.9109	0.7552	0.4984

## Data Availability

The original data presented in the study are openly available at [22].
